# Supplementary material for: End Sequence Analysis Toolkit (ESAT) expands the extractable information from single-cell RNA-seq data
Source: Genome Res. 2016 Oct;26(10):1397–410. doi: 10.1101/gr.207902.116 (PMC5052061; doi:10.1101/gr.207902.116)
Supplement: Supplemental Material [file supp_gr.207902.116_Supp_Materials.tar.gz › ESAT-esat_v0.1/doc/html/dir_e2964a2d1e4eec4529e25374aaf11fed.html]

ESAT: java/broad/core/siphy Directory Reference


ESAT


- Main Page
- Related Pages
- Packages
- Classes
- Files

All Classes Namespaces Files Functions Variables Enumerator Pages

- java
- broad
- core
- siphy

siphy Directory Reference

Directory dependency graph for siphy:

|  |  |
| --- | --- |
| Directories | |
| directory | tools |
|  | |

|  |  |
| --- | --- |
| Files | |
| file | ConservationUtils.java |
|  | |
| file | ConservedPWMScanner.java |
|  | |
| file | EvolutionaryModel.java |
|  | |
| file | EvolutionaryModelParameters.java |
|  | |
| file | Fit.java |
|  | |
| file | PhastconsReader.java |
|  | |
| file | PiHMM.java |
|  | |
| file | PositionWeightMatrixModel.java |
|  | |
| file | ScaledWindowParserListener.java |
|  | |
| file | StationaryDistributionIO.java |
|  | |
| file | TreeScaler.java |
|  | |
| file | TreeScalerIO.java |
|  | |
| file | UnableToFitException.java |
|  | |


---

Generated on Fri Aug 15 2014 15:11:16 for ESAT by  

 1.8.7
